# Early-life epigenetic programming of autoimmune diseases: what is unique to type 1 diabetes

**DOI:** 10.3389/fimmu.2026.1946981

**Published:** 2026-10-21

**Authors:** Shamila D. Alipoor, Julia Åkesson, Johnny Ludvigsson

**Affiliations:** 1 Division of Pediatrics, Department of Biomedical and Clinical Sciences, Linköping University, Linköping, Sweden; 2 Division of Bioinformatics, Department of Physics, Chemistry and Biology, Linköping University, Linköping, Sweden; 3 PredictMe AB, Linköping, Sweden; 4 Crown Princess Victoria Children’s Hospital, Linköping University Hospital, Linköping, Sweden

**Keywords:** autoimmune diseases, early-life epigenetic programming, epigenomics, proteomics, type 1 diabetes

## Abstract

**Introduction:**

Type 1 diabetes (T1D) is an autoimmune disease characterized by progressive pancreatic β-cell destruction, yet molecular features distinguishing it from other autoimmune conditions in early life remain poorly defined. We previously identified neonatal proteomic signatures predicting future T1D years before diagnosis, suggesting immune and tissue-remodeling pathways are already perturbed at birth. However, whether these alterations reflect a general autoimmune predisposition or are specific to T1D has remained unclear.

**Method:**

To address this, we integrated cord blood DNA methylation profiles with neonatal serum proteomic data from the All Babies in Southeast Sweden (ABIS) cohort and compared T1D with other autoimmune diseases except T1D (NT1D).

**Result:**

In an exploratory analyse, we observed a distinct early-life molecular pattern in T1D, most consistently characterized by dysregulation of β-cell metabolic genes and a CD40-centered co-stimulatory immune signal. Several components of a previously reported neonatal proteomic signature of future T1D were re-evaluated in the context of NT1D. HLA-related antigen presentation and related immune-activation signals recurred in the NT1D associated networks as well, suggesting these largely reflect broader autoimmune biology, whereas co-stimulatory signaling (CD40) and β-cell metabolic pathways showed stronger representation in T1D-associated analyses. In contrast, the NT1D profile was characterized predominantly by broader interferon-γ-associated and chemokine-driven inflammatory signaling.

**Conclusion:**

These findings identify an exploratory early-life 43,epigenetic–proteomic pattern in T1D that combines immune and β-cell-related metabolic features, while also highlighting molecular signals that may be shared with other autoimmune conditions. These observations support further investigation of early-life molecular differences between T1D and other autoimmune diseases in larger, independent cohort.

## 1 Introduction

Type 1 diabetes (T1D) is a heterogeneous and polygenic autoimmune disease in which genetic background, environmental exposures, and immune dysregulation converge to drive progressive pancreatic β-cell destruction (1). While autoimmune diseases share a broad landscape of immune dysfunction, each condition exhibits organ specificity. T1D is characterized by immune-mediated targeting of insulin-producing β-cells, distinguishing it from other autoimmune disorders not only by its tissue specificity but also by its early-life onset, metabolic consequences, and heterogeneous genetic architecture (2).

Unlike chronic inflammatory conditions such as rheumatoid arthritis or relapsing–remitting diseases such as multiple sclerosis (MS), T1D typically involves rapid and irreversible loss of β-cell mass, leading to absolute insulin deficiency and lifelong dependence on exogenous insulin therapy (3).

Although T1D is considered an organ-specific autoimmune disease, it frequently coexists with other autoimmune conditions (3, 4). Up to one-third of individuals with T1D develop additional autoimmune diseases (5, 6), most commonly autoimmune thyroid disorders (7), but also including celiac disease (8), autoimmune gastritis, and Addison’s disease (4). This clustering suggests shared underlying mechanisms; however, the selective vulnerability of β-cells remains poorly understood.

Genetically, the strongest risk for T1D is conferred by variants within the human leukocyte antigen (HLA) region (9). Although approximately 25-30% of the Swedish general population carries T1D-associated HLA risk alleles, only less than 2% ever develop the disease, and a substantial proportion of T1D cases arises in individuals without high-risk HLA alleles (10) further underlining that genetic predisposition alone is insufficient to explain disease onset. This is also supported by the relatively low concordance rate (~40%) observed in monozygotic twins, underscoring the critical contribution of non-genetic factors (9).

Epigenetic modifications, such as DNA methylation, provide a dynamic interface between the genome and environmental influences, regulating gene expression without altering the underlying DNA sequence (11). Epigenome-wide association studies (EWAS) have identified disease-associated methylation changes at key immune and metabolic loci, including the insulin (*INS*) gene (12, 13) and *IL2RA* (13, 14). Altered methylation patterns have also been observed in regulatory T cells, particularly within the *FOXP3* locus, potentially impairing immune tolerance and promoting autoimmune responses (15).

Early life represents a critical window during which epigenetic programming may shape long-term immune function and disease susceptibility (16). Prenatal and perinatal exposures have been shown to induce persistent epigenetic modifications, as illustrated by the reduced risk of T1D observed in offspring of mothers with T1D (17, 18). Our recent analyses of cord blood DNA from the ABIS birth cohort (19, 20) revealed distinct methylation signatures in individuals who later developed T1D, dependent on HLA risk status, suggesting heterogeneity in early-life epigenetic programming (13). In a separate proteomic study of the same birth cohort, we recently reported a neonatal inflammatory signature that predicted future T1D years before diagnosis independently of HLA. The altered proteins were enriched in pathways related to neutrophil migration, cytotoxicity, extracellular matrix remodeling, and immune regulation, suggesting that immune and tissue-remodeling processes are already perturbed at birth (21). These findings raised the question of whether these circulating protein differences reflect a general predisposition to autoimmunity or a molecular program specific to T1D, and whether they are rooted in epigenetic alterations established at birth (21).

Here, we sought to address this question by investigating whether individuals who later develop T1D exhibit early-life molecular features that are more strongly associated with T1D than with other autoimmune conditions. To this end, we integrated cord blood DNA methylation profiles with neonatal serum proteomic data from the ABIS birth cohort. Through this integrative approach, we identified an exploratory early-life epigenetic–proteomic molecular profile associated with T1D, comprising both shared autoimmune features and molecular features more strongly represented in T1D.

## 2 Materials and methods

### 2.1 Data source and study design

This study is a secondary analysis of a cord blood DNA methylation dataset from the All Babies in Southeast Sweden (ABIS) cohort (19, 20), previously described in detail (13). Cord blood DNA and serum samples were collected at birth from all participants. Diagnoses of T1D were obtained from the Swedish National Patient Register and validated using data from the National Drug Prescription Register, yielding a total of 168 T1D cases. Among them we selected those HLA-typed individuals with available DNA samples, meaning 32 with T1D, as well as individuals with other autoimmune diseases excluding T1D (NT1D, n=35) identified using ICD-10 codes which comprised celiac disease (K90.0, n=19), psoriasis (L40, n=5), autoimmune thyroiditis (E06.3, n=4), Crohn’s disease (K50, n=3), juvenile idiopathic arthritis (M08, n=2), and uveitis (H20, n=2). A total of 48 individuals with no recorded history of autoimmune or immune-mediated disease were included as controls. Sex was included as a covariate in all differential methylation analyses. Participants were diagnosed with T1D at a median age of 11.4 years, and 75% were positive for at least one islet autoantibody (GADA, IA-2A, IAA, or ZnT8), at diagnosis.

### 2.2 Ethics

As previously reported, the ABIS study was approved by the Research Ethics Committees of the Faculty of Health Sciences, Linköping University (Ref. 1997/96287 and 2003/03-092) and the Medical Faculty of Lund University (Dnr 99227, Dnr 99321). Linkage to national registers was approved by the Swedish Data Inspection Board (Dnr 03-513 and 2018-318-32). Written informed consent was obtained from parents or legal guardians. The current secondary analysis was conducted under these approvals.

### 2.3 DNA extraction and methylation profiling

DNA concentration and purity were determined using fluorescence-based quantification and spectrophotometric assessment. For methylation profiling, 250 ng of genomic DNA per sample was subjected to bisulfite conversion using the EZ DNA Methylation Kit (Zymo Research). Genome-wide methylation patterns were subsequently assessed using the Illumina Infinium MethylationEPIC v1.0 BeadChip platform, with array scanning performed on an Illumina NextSeq 550 instrument. Raw signal intensity data in IDAT format were retained for all downstream bioinformatic processing.

### 2.4 Preprocessing, QC, and DMG definition

Data preprocessing and quality control were conducted following the analytical pipeline detailed in our previous publication (13). In brief, raw methylation intensities were imported into R and processed using the minfi package. Intra-sample normalization was carried out using the Beta-Mixture Quantile (BMIQ) method, followed by inter-array normalization using Gaussian Mixture Quantile Normalization (GMQN). Probe-level filtering excluded probes with missing values exceeding 25% of samples, non-CpG probes, cross-reactive probes, probes overlapping known single nucleotide polymorphisms, and probes located on sex chromosomes. Technical batch effects were identified through singular value decomposition (SVD) analysis and corrected using the ComBat algorithm, with successful artifact removal confirmed by inspection of beta-value density distributions and principal component analysis. Differential methylation between groups was assessed using linear regression models implemented in the limma package, with sex and significantly differing cell type proportions included as covariates. A CpG site was considered differentially methylated when both the nominal p-value was below 0.05 and the absolute log fold change exceeded 0.05 (P ≤ 0.05 and |logFC| ≥ 0.05). Differentially methylated CpG sites (DMCs) were subsequently mapped to their corresponding genes using Illumina MethylationEPIC array annotations based on the hg19 genome assembly, yielding lists of differentially methylated genes (DMGs) for each group comparison.

### 2.5 Pathway enrichment analysis

KEGG pathway enrichment analysis of differentially methylated CpGs was performed using the probe-aware gometh function implemented in the missMethyl R package. The post-quality-control CpGs that entered the differential methylation analyses were used as the background probe set. The Wallenius noncentral hypergeometric test was used to account for differences in probe numbers across genes. The analysis was performed with fract.counts = TRUE to account for CpGs annotated to multiple genes. CpGs meeting the nominal significance threshold of P ≤0.05 and |Δβ|≥ 0.05 were used as the significant input set.

### 2.6 Serum proteomics and statistical analysis

Circulating protein levels in neonatal serum were measured using the Olink Explore 384 Inflammation and Oncology panels (Olink Proteomics, Uppsala, Sweden), deployed on the next-generation sequencing-based Olink Explore platform (22). Protein abundance was expressed as Normalized Protein eXpression (NPX) values on a log2 scale, covering 727 assays following manufacturer-specified quality control procedures and normalization protocols. Differences in protein abundance between groups were assessed using linear models implemented in the limma package, with sex and pool included as covariates, followed by empirical Bayes moderation of variance estimates. Proteins were considered differentially abundant at a nominal P-value ≤ 0.05 and an absolute median ΔNPX ≥ 0.2.

### 2.7 Integration and network analysis of epigenetic-proteomic data

To investigate cross-layer molecular interactions, differentially methylated genes (DMGs) from cord blood DNA methylation data and differentially abundant serum proteins were integrated using a network-based approach. DMGs were obtained from our previously published epigenome-wide analysis of the ABIS cohort (19, 20), in which differential methylation was assessed using the limma R package (23). CpG sites were considered differentially methylated at nominal p-value thresholds (P ≤ 0.05) and |logFC| > 0.05. The resulting list of DMGs was used as input for the integrative network analysis. Serum proteins were selected based on effect size (|ΔNPX| ≥ 0.2) and nominal p ≤ 0.05.

The molecular inputs for network analysis were derived from the quality-controlled methylation and proteomic datasets. The proteomic component comprised the 727 Olink assays that passed quality control, while the methylation component comprised CpG sites retained after preprocessing and quality control, from which DMGs were derived for network construction. The resulting DMGs and differentially abundant proteins from each comparison were combined and mapped to the STRING database (version 12.0) (24) for protein–protein interaction (PPI) retrieval. Unmapped identifiers were excluded (removeUnmappedRows = TRUE). Only high-confidence interactions (combined score ≥ 0.7) were retained. The resulting integrated epigenetic–proteomic interaction network was constructed separately for each comparison.

## 3 Results

Differential DNA methylation analysis was performed using the analytical workflow described in our previous epigenome-wide study of the ABIS cohort ([8]). Differential proteomic analysis of neonatal Olink data identified multiple significantly altered serum proteins across the study comparisons. The complete proteomic results, including differentially abundant proteins, KEGG pathway enrichment analyses, and representative boxplots, are provided in **Supplementary File 1**. The resulting differentially methylated genes (DMGs) and differentially abundant proteins were subsequently integrated using protein–protein interaction network analysis to investigate epigenetic–proteomic molecular interactions across the comparison groups.

### 3.1 Epigenetic and proteomic landscape in T1D compared to other autoimmune diseases

To identify molecular features specific to T1D relative to other autoimmune diseases (NT1D), we performed methylation profiling of cord blood DNA collected at birth from individuals who later developed T1D and compared them with individuals who later developed NT1D.

Differential methylation analysis identifies a set of significantly dysregulated CpG sites. Representative differentially methylated genes in T1D compared to NT1D included PIK3CD, TLR3, NFATC1, ENO1, and GCK (**Figure 1A**).

**Figure 1:**
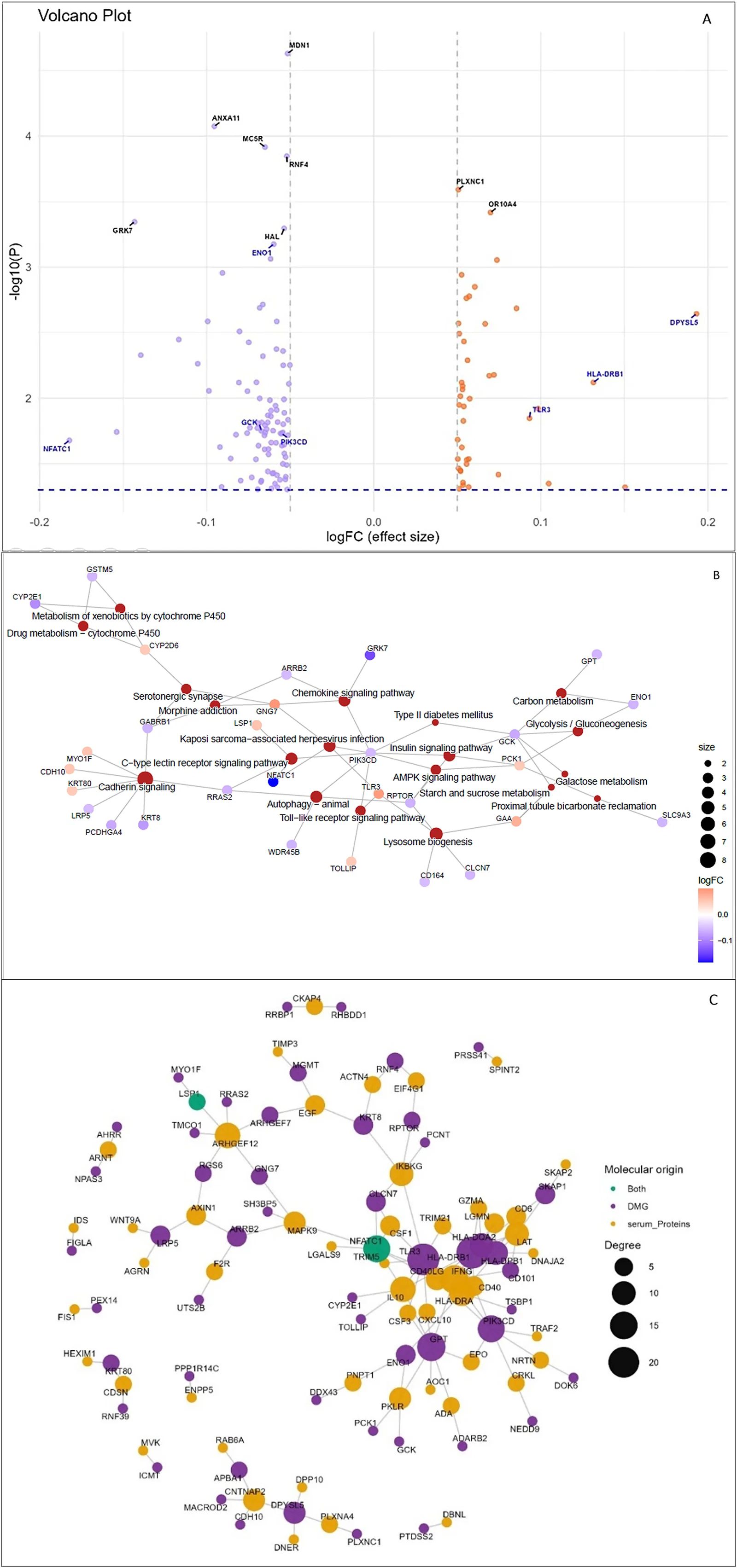
Epigenetic and proteomic molecular landscape in T1D compared to other autoimmune diseases (NT1D) at birth. **(A)** Volcano plot of differentially methylated genes (DMGs) identified from cord blood DNA comparing individuals who later developed T1D with those who developed other autoimmune diseases (NT1D), with logFC on the x-axis and −log10(p-value) on the y-axis, with positive logFC indicating hypermethylation and negative logFC indicating hypomethylation in T1D relative to NT1D. Representative DMGs selected based on biological relevance are labeled in the volcano plot **(B)** KEGG concept network plot (cnetplot) showing connections between enriched pathways and their associated DMGs, with gene nodes colored by logFC direction (red: hypermethylated in T1D; blue: hypomethylated in T1D). **(C)** Integrated epigenetic–proteomic interaction network of T1D vs NT1D, with nodes colored by molecular origin (purple: DMG; orange: serum protein; green: both). This network was constructed using all seven STRING v12 evidence channels including automated text mining of the literature. A sensitivity analysis excluding the text-mining evidence channel is provided in **Supplementary Table 3**. Features were selected using a nominal P-value threshold of ≤0.05.

KEGG pathway enrichment analysis of the identified DMGs (**Figure 1B**) revealed two major functional clusters. A metabolic cluster comprised pathways related to glycolysis and gluconeogenesis, carbon metabolism, insulin signaling, AMPK signaling, Type II diabetes mellitus, galactose metabolism, and starch and sucrose metabolism. These pathways were primarily connected through GCK, ENO1, PCK1, RPTOR, and PIK3CD. In addition, autophagy- and lysosome-related pathways were enriched among the identified DMGs.

The second cluster consisted mainly of structural and adhesion-related pathways and included genes such as CDH10, KRT8, and LRP5 associated with cadherin signaling. Several genes involved in neurodevelopmental and neuro-immune processes, including GABRB1 were also identified among the differentially methylated genes (**Figure 1B**).

To further investigate molecular interactions distinguishing T1D from NT1D, we performed an integrative epigenetic–proteomic interaction network analysis combining DMGs with serum proteomic profiles (**Figure 1C**). This analysis revealed a highly interconnected network characterized by prominent immune-related hubs, including HLA class II molecules (HLA-DRA, HLA-DRB1, and HLA-DQA2). Additional central network nodes included PIK3CD, IFNG, IL10, CD40, and CD40LG. Differentially methylated antiviral and innate immune-related genes, including TRIM5 and TRIM21, were also identified within the interaction network. Furthermore, several genes involved in glucose metabolism and cellular energy regulation, including GCK, PKLR, PCK1, and ENO1, were connected to serum proteins within the integrated network. Genes associated with neurodevelopmental and neuro-immune pathways, including DPYSL5, ADARB2, and NPAS3, were also represented in the interaction network. LSP1 appeared as a shared node across both epigenetic and proteomic layers, representing both a differentially methylated gene and a serum protein. Additional genes related to insulin and glucose homeostasis and stress responses, including ARHGEF12 and NFATC1, were identified within the interaction network (**Figure 1C**).

### 3.2 Epigenetic and proteomic landscape of NT1D relative to controls

To distinguish features more strongly represented in T1D from those shared across autoimmune conditions, in the next step we compared the epigenetic and proteomic landscape of NT1D individuals with healthy controls. Differential methylation analysis revealed a sparse set of significantly dysregulated CpG sites compared to the T1D vs NT1D comparison, with smaller effect sizes across the volcano plot (**Figure 2A**). Representative hypomethylated genes in NT1D included PRKCE, RPTOR, while hypermethylated genes included TIMP2, BLK, CD101, ERCC6, HDAC4,and KIF1B (**Figure 2A**).

**Figure 2:**
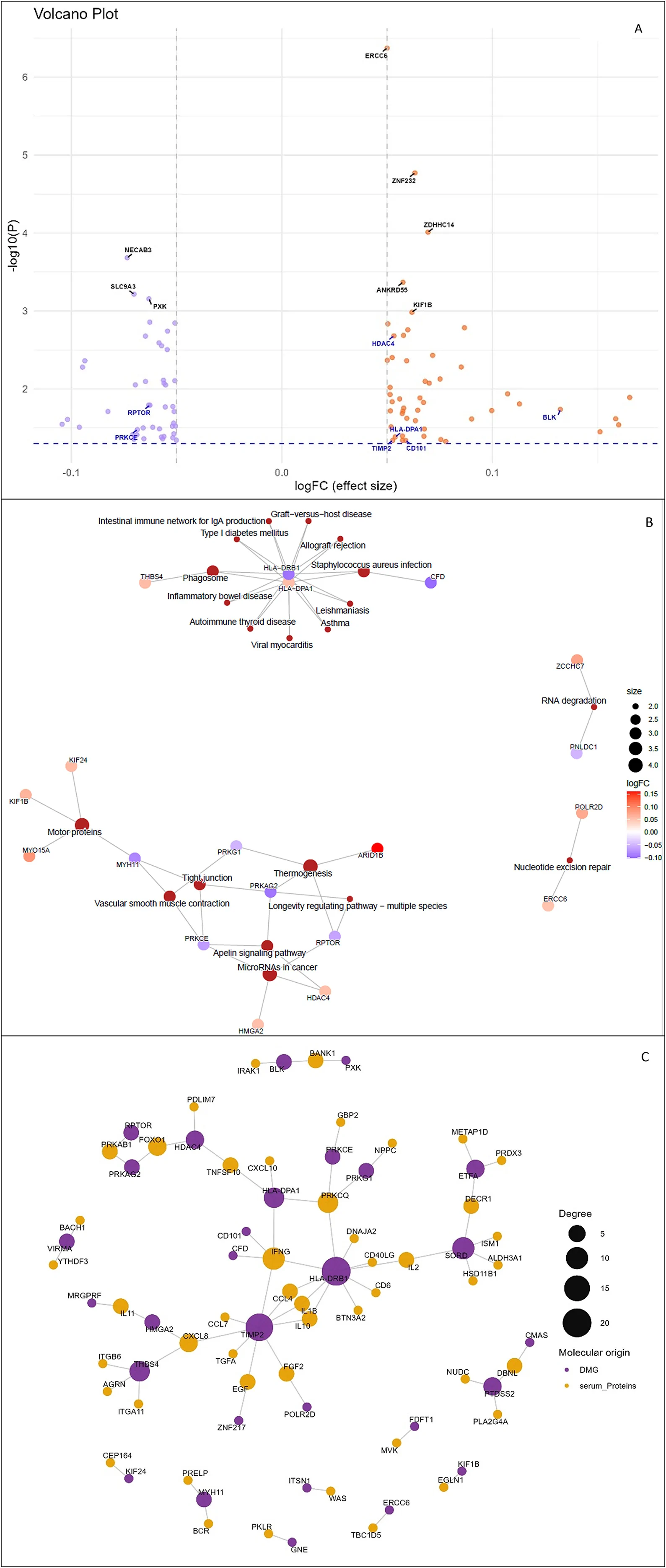
Epigenetic and proteomic molecular landscape of other autoimmune diseases relative to healthy controls at birth. **(A)** Volcano plot of differentially methylated genes (DMGs) from cord blood DNA comparing NT1D individuals with healthy controls, with logFC on the x-axis and −log10(p-value) on the y-axis, positive logFC indicating hypermethylation and negative logFC indicating hypomethylation NT1D relative to controls. Representative DMGs selected based on biological relevance are labeled in the volcano plot **(B)** KEGG concept network plot showing connections between enriched pathways and associated DMGs, with gene nodes colored by logFC direction and sized by the number of pathway connections. **(C)** Integrated epigenetic-proteomic interaction network of NT1D vs healthy controls, with nodes colored by molecular origin (purple: DMG; orange: serum protein) and sized by network degree. This network was constructed using all seven STRING v12 evidence channels including automated text mining of the literature. A sensitivity analysis excluding the text-mining evidence channel is provided in **Supplementary Table 3**. Features were selected using a nominal P-value threshold of ≤0.05.

KEGG pathway enrichment of NT1D-associated DMGs revealed a predominantly HLA-driven immune cluster associated with broad autoimmune and inflammatory pathway enrichment (**Figure 2B**). Additional enrichment was observed for thermogenesis and motor protein-related pathways connected through kinase and cytoskeletal genes including PRKAG2, PRKCE, RPTOR, KIF1B, KIF24, and MYH11. A separate chromatin remodeling-associated cluster involving HDAC4, HMGA2, and ARID1B were also identified. In contrast to T1D-associated analyses, classical β-cell metabolic genes including GCK, ENO1, PCK1, and PKLR were not represented among NT1D-associated DMGs or enriched pathway (**Figure 2B**).

To provide context for disease-specific features, we next examined the integrative interaction network in NT1D compared to controls (**Figure 2C**). In the NT1D versus control network, TIMP2 was identified as a prominent proteomic hub (hypermethylated/lower expression in NT1D patients compared to controls), reflecting processes related to extracellular matrix remodeling, which are commonly observed across autoimmune diseases. In parallel, IFNγ (IFNG) appeared as a prominent serum protein hub, indicating that interferon-γ-related signaling may represent a shared feature of autoimmune immune activation (**Figure 2C**).

A pronounced chemokine signature comprising CXCL8, CCL4, and CCL7 was prominent in the NT1D network, reflecting broad inflammatory cytokine recruitment characteristic of the general autoimmune landscape. PRKCQ, a T cell-specific kinase involved in NF-κB and AP-1 signaling, along with chromatin regulators HMGA2, HDAC4, and FOXO1, and AMPK subunits PRKAB1 and PRKAG2, further characterized the NT1D network as reflecting heterogeneous immune regulatory interaction patterns shared across autoimmune diseases.

### 3.3 Epigenetic and proteomic landscape of T1D relative to controls at birth

The T1D versus control comparison identified a set of differentially methylated CpG sites. Representative differentially methylated genes (DMGs) are visualized in the volcano plot (**Figure 3A**), highlighting a composite molecular profile characterized by immune activation, metabolic regulation, and neurodevelopmental features that were more strongly represented in T1D-associated analyses. Among the significantly differentially methylated genes in T1D was FGFR2 encoding fibroblast growth factor receptor 2, alongside GCK, PRKCE, BLK and MBP.

**Figure 3:**
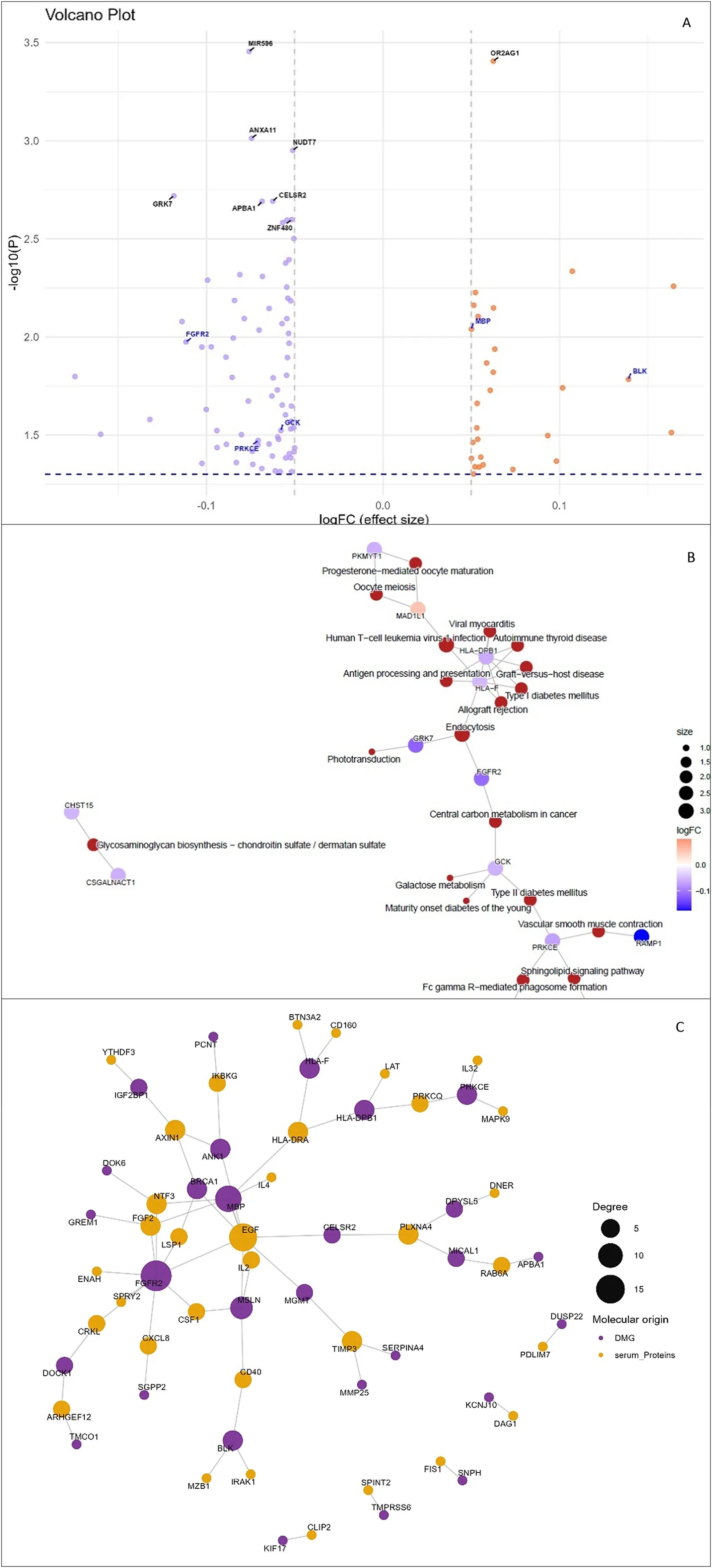
Epigenetic and proteomic molecular landscape of Type 1 diabetes relative to healthy controls at birth. **(A)** Volcano plot of differentially methylated genes (DMGs) from cord blood DNA comparing T1D individuals with healthy controls, with logFC on the x-axis and −log10(p-value) on the y-axis., Positive logFC indicating hypermethylation and negative logFC indicating hypomethylation T1D relative to controls. Representative DMGs selected based on biological relevance are labeled in the volcano plot **(B)** KEGG concept network plot showing connections between enriched pathways and associated DMGs, with gene nodes colored by logFC direction and sized by the number of pathway connections. **(C)** Integrated epigenetic-proteomic interaction network of T1D vs healthy controls, with nodes colored by molecular origin (purple: DMG; orange: serum protein) and sized by network degree. This network was constructed using all seven STRING v12 evidence channels including automated text mining of the literature. A sensitivity analysis excluding the text-mining evidence channel is provided in **Supplementary Table 3**. Features were selected using a nominal P-value threshold of ≤0.05.

KEGG pathway enrichment of T1D DMGs revealed a unique co-enrichment of both the Type I diabetes and T2D pathways (**Figure 3B**). The T1D pathway was driven by HLA-DPB1 and HLA-F as central connector genes in the cnetplot, linking antigen processing and presentation, allograft rejection, graft-versus-host disease, autoimmune thyroid disease, and viral myocarditis in an HLA-centered immune cluster. The T2D pathway was driven by GCK connecting to galactose metabolism, maturity onset diabetes of the young (MODY), and central carbon metabolism in cancer, forming a distinct metabolic cluster entirely separate from the immune cluster. Glycosaminoglycan biosynthesis chondroitin sulfate/dermatan sulfate, driven by CHST15 and CSGALNACT1, reflects extracellular matrix remodeling relevant to the islet microenvironment. Additional pathway enrichments included FGFR2 connecting endocytosis and central carbon metabolism, and PRKCE connecting vascular smooth muscle contraction, sphingolipid signaling, and Fc gamma receptor-mediated phagosome formation.

Integrative epigenetic-proteomic interaction network analysis of T1D vs healthy controls (**Figure 3C**) identified EGF and MBP as the dominant serum protein hubs, with FGFR2 as the largest DMG hub, consistent with its top-ranked position in the volcano plot (**Figure 3A**) and its connection to growth factor and metabolic pathways in the cnetplot (**Figure 3B**). BLK emerged as a major DMG hub connected to MZB1, IRAK1, and CD40, reinforcing the B cell activation axis identified in the methylation analysis. IL2, IL4, and CSF1 appeared as cytokine connecting nodes, while a neurodevelopmental cluster comprising DPYSL5, DNER, and PLXNA4 was identified on the network periphery. LSP1 appeared as a DMG in this network, further supporting its consistent presence across T1D comparisons.

## 4 Discussion

We have previously shown, using Olink proteomic analyses of cord blood samples from the ABIS cohort, that inflammatory signals are differentially abundant years before the diagnosis of T1D. Children who later developed T1D displayed a detectable inflammatory proteomic signature at birth, characterized by increased HLA-DRA, IDS, and CTSC and decreased TIMP3, ADA, and CD40LG. These proteins were enriched in pathways related to neutrophil migration, cytotoxicity, extracellular matrix remodeling, and immune regulation, and a subset of these markers predicted future T1D with high accuracy (AUC = 0.89 ± 0.02) (25). Those findings provided evidence that the path toward T1D may begin *in utero*, involving early abnormalities in innate immunity, antigen presentation, and tissue remodeling (25). However, it remained unclear whether these early molecular signatures reflected a general predisposition to autoimmunity or represented developmental processes more strongly associated with future T1D. The present study addresses this gap by integrating neonatal proteomic profiles with cord blood DNA methylation data and directly comparing T1D with other autoimmune diseases.

Our findings identify an exploratory early-life epigenetic–proteomic pattern in T1D that includes both immune and β-cell-related metabolic features. Comparison with NT1D showed that some immune and inflammatory signals were shared across autoimmune conditions, whereas β-cell-related metabolic features and co-stimulatory immune signals were more strongly represented in T1D.

A particularly notable finding specific to T1D group was the differential methylation of β-cell-related metabolic genes, including GCK, ENO1, PKLR, and PCK1 in T1D group. GCK and ENO1 encode glucokinase and enolase 1, respectively, both core enzymes in glucose sensing and glycolytic metabolism in pancreatic β-cells (29), while PKLR and PCK1 further implicate pyruvate kinase and gluconeogenic regulation (30). Their representation in the T1D associated networks, provides comparatively stronger support for a β-cell-related metabolic component early life presented in T1D than in NT1D (**Table 1**).

**Table 1:** Key molecular features across the three pairwise comparisons.

Molecular feature	NT1D vs control	T1D vs NT1D	T1D vs control	Interpretation
HLA-associated antigen presentation	✓	✓	✓	shared autoimmune feature
Co-stimulatory/T-cell signaling	✓	✓	✓	Candidate shared immune-activation feature
B-cell-associated signaling	✓	✓	✓	Candidate shared immune-regulatory feature (BLK/IRAK1-related signaling represented across all three comparisons)
Inflammatory cytokine/chemokine signaling	✓	✓	–	Shared inflammatory process with different cytokine/chemokine composition between groups
IFNG-associated signaling	✓	✓	–	NT1D-enriched inflammatory feature
Extracellular-matrix remodeling	✓	✓	✓	Tissue-remodeling process with distinct molecular hubs in each group
Glucose and central carbon metabolism	–	✓	✓	T1D-enriched metabolic feature
Neuronal-related processes	–	✓	✓	T1D-enriched neurodevelopmental feature
Autophagy/lysosome and xenobiotic metabolism	–	✓	✓	Disease-discriminatory features; direction cannot be assigned from the intergroup comparison alone

Additional metabolic findings further supported the presence of altered metabolic processes in T1D-associated analyses. FGFR2, which encodes fibroblast growth factor receptor 2 and plays roles in tissue repair, immune modulation, and β-cell survival and proliferation, was among the most significantly hypermethylated DMGs in T1D. Its network connectivity to serum protein hubs including EGF, FGF2, NTF3, and CSF1 suggests coordinated involvement of growth-factor signaling in the early molecular profile associated with T1D. Consistently, enrichment of insulin signaling and Type II diabetes pathways among T1D-associated DMGs suggests overlap between T1D-associated epigenetic findings and metabolic dysregulation, consistent with our previously reported T2D-like signatures in low HLA-risk T1D developers (13). These observations suggest that metabolic dysregulation may form part of the early molecular context associated with T1D, although the mechanisms underlying these associations remain to be determined.

Extracellular-matrix remodeling was represented across all three comparisons, but with different molecular features in the two autoimmune groups. TIMP2 was prominent in the NT1D-associated network, whereas TIMP3 was represented in the T1D-associated networks. Thus, although extracellular-matrix remodeling may reflect a broader autoimmune-associated process, the specific molecular components involved may differ between T1D and other autoimmune diseases. Notably, TIMP3 was also reduced in neonatal serum of children who subsequently developed T1D in our previous proteomic study (25), providing independent support within our work for its association with early-life T1D-related molecular alterations. Given the established role of TIMP3 in tissue integrity and inflammatory regulation, this finding is consistent with a possible contribution of tissue-remodeling processes to early T1D-associated molecular alterations. Experimental evidence indicates that TIMP3 supports islet integrity and limits inflammatory signaling, suggesting that reduced TIMP3 at birth may compromise the structural and anti-inflammatory protection of the islet microenvironment long before autoimmune activation (25, 26). Consistent with this interpretation, pathways related to extracellular matrix remodeling and glycosaminoglycan biology were also represented among the T1D-associated findings at the epigenetic level. Given the critical role of extracellular matrix integrity in maintaining islet structure, regulating immune-cell infiltration, and supporting β-cell homeostasis (27, 28), our findings raise the possibility that altered tissue-remodeling processes may represent an early component of the molecular profile associated with T1D. This hypothesis will require confirmation in adequately powered, independent cohorts.

We observed that HLA-related antigen-presentation features were prominent in both T1D and the NT1D related networks. However, T1D-associated network contained a prominent co-stimulatory immune component involving CD40 and CD40LG, together with PIK3CD and other immune-regulatory nodes. CD40–CD40LG signaling has established roles in T-cell and B-cell interactions and has previously been implicated in autoimmune diabetes (34–36). Notably, CD40 was represented in the T1D-associated networks but was not observed in the NT1D versus control network, whereas CD40LG was represented across all three comparisons. This pattern suggests that CD40-associated co-stimulatory signaling may be more strongly represented in T1D than in NT1D, while CD40LG may reflect a broader autoimmune-associated signal (**Table 1**).

LSP1, previously implicated in neutrophil migration pathways in our earlier proteomic signature (25), showed the same pattern as CD40, represented in both T1D-containing comparisons but absent from the NT1D-versus-control network, providing additional support for its association with T1D specifically.

In contrast, the NT1D analysis was characterized predominantly by broader inflammatory and autoimmune-associated signals. HLA-related antigen-presentation pathways were prominent, while IFNG and chemokines including CXCL8, CCL4, and CCL7 were represented in NT1D-associated interaction networks. This specific chemokine pattern was not prominent in the T1D-associated networks.

The predominance of broad, innate-recruiting chemokines (CXCL8, CCL4, CCL7) in the NT1D networks, contrasted with the antigen-presentation and co-stimulatory features more prominent in the T1D-associated networks, may reflect a broader distinction between generalized innate inflammatory activation in NT1D and a more targeted, antigen-directed adaptive immune process in T1D, consistent with the organ-specific, T-cell-mediated pathogenesis classically described for T1D (**Table 1**). However, given that the NT1D group comprises a heterogeneous set of autoimmune conditions, this pattern should be interpreted cautiously and requires confirmation in larger disease-matched cohorts.

Additional features observed in T1D-associated analyses included genes involved in neurodevelopmental and neuro-immune processes. DPYSL5, ADARB2, NPAS3, and GABRB1 were among the genes represented in these analyses, and GABRB1 was identified as a hypomethylated DMG in the T1D versus NT1D comparison. Similar neuro-related signals were also observed in our previous work, suggesting that neurodevelopmental or neuro-immune pathways may warrant further investigation in the context of early-life molecular alterations associated with T1D (13, 47). However, these findings should be interpreted as exploratory rather than as evidence for a primary neurodevelopmental mechanism in T1D.

A smaller set of additional features, including structural/adhesion genes (CDH10, KRT8, KRT80, LRP5), innate antiviral sensing genes (TRIM5, TRIM21), and enrichment of autophagy and lysosome biogenesis pathways consistent with β-cell endoplasmic reticulum stress (13, 31), were represented in the T1D-versus-NT1D comparison. BLK, previously implicated in diabetes susceptibility (32, 33) and connected to MZB1, IRAK1, and CD40 in the present networks, was represented in both the T1D-versus-control and NT1D-versus-control comparisons and is therefore more likely to reflect shared B-cell-related autoimmune signaling than a T1D-specific feature.

Taken together, our observation indicates that the early-life molecular profile previously associated with future T1D comprises a substantial shared autoimmune component including HLA-related antigen presentation, CD40LG, and BLK-related B-cell signaling together with a smaller set of features showing stronger support for T1D specificity, most notably the β-cell-related metabolic genes (GCK, ENO1, PKLR, PCK1), CD40, and LSP1.

Rather than indicating a single disease-specific pathway, these findings support a model in which a limited set of β-cell metabolic and co-stimulatory immune features may distinguish the early-life molecular profile of T1D from other autoimmune conditions, within a broader shared autoimmune context. Importantly, the integrated networks represent known molecular interactions among study-derived features rather than direct individual-level methylation–protein covariation, and regarding the small size of our cohort most differences did not survive multiple-testing correction; the findings should therefore be interpreted as an exploratory molecular framework generating hypotheses for future longitudinal and multi-omics studies.

## 5 Study limitation

Several limitations should be considered when interpreting the present findings. First, the study was conducted in a relatively modest-sized cohort, which may have limited statistical power, particularly for the DNA methylation analyses where relatively few CpG sites reached statistical significance after multiple-testing correction. Accordingly, the identified differentially methylated CpGs (DMCs), their corresponding differentially methylated genes (DMGs), and the downstream pathway and network analyses should be considered exploratory and hypothesis-generating, requiring validation in larger independent cohorts.

Second, the study lacks an independent external validation cohort. The ABIS cohort is unique in its prospective population-based design, and availability of both cord blood DNA methylation and neonatal proteomic data, and no comparable cohort is currently available for direct replication. Nevertheless, independent validation will be essential to confirm the robustness and generalizability of the present findings.

Third, the non-type 1 diabetes (NT1D) group comprised a heterogeneous collection of autoimmune diseases with distinct immunopathogenic mechanisms. Although this design enabled us to investigate molecular features associated with autoimmunity beyond T1D, the biological heterogeneity of the comparison group may have influenced the specificity of the observed molecular differences. Future studies including larger cohorts of individual autoimmune diseases will be important to determine whether the identified molecular signatures are truly specific to T1D or reflect disease-specific characteristics within the comparison group.

## 6 Conclusion

In conclusion, this study identifies an exploratory early-life epigenetic–proteomic pattern associated with future T1D that combines immune dysregulation with β-cell-related metabolic features. Comparison with NT1D indicates that some immune and inflammatory signals, including HLA-related antigen presentation are shared across autoimmune conditions, whereas β-cell-related metabolic features and selected co-stimulatory immune signals are more strongly represented in T1D-associated analyses. Additional tissue-remodeling, growth-factor, neurodevelopmental, and cellular stress-response features further broaden the molecular profile observed in T1D. These findings support a model in which multiple early-life biological processes may contribute to later T1D development. However, given the exploratory nature of the analyses, these findings should be considered hypothesis-generating and require validation in larger, independent longitudinal studies.

## Data Availability

The datasets presented in this article are not readily available because of ethical considerations and participant confidentiality. Requests to access the datasets should be directed to the corresponding author, Johnny Ludvigsson (johnny.ludvigsson@liu.se), and access is subject to ethical approval.

## References

[1] Rewers M Ludvigsson J . Environmental risk factors for type 1 diabetes. Lancet. (2016) 387:2340–8. doi: 10.1016/s0140-6736(16)30507-4 27302273 PMC5571740

[2] Kawasaki E . Type 1 diabetes and autoimmunity. Clin Pediatr Endocrinol. (2014) 23:99–105. doi: 10.1297/cpe.23.99 25374439 PMC4219937

[3] Popoviciu MS Kaka N Sethi Y Patel N Chopra H Cavalu S . Type 1 diabetes mellitus and autoimmune diseases: A critical review of the association and the application of personalized medicine. J Personalized Med. (2023) 13:422. doi: 10.3390/jpm13030422 36983604 PMC10056161

[4] Nederstigt C Uitbeijerse B Janssen L Corssmit E de Koning E Dekkers O . Associated auto-immune disease in type 1 diabetes patients: A systematic review and meta-analysis. Eur J Endocrinol. (2019) 180:135–44. doi: 10.1530/eje-18-0515 30508413

[5] Triolo TM Armstrong TK McFann K Yu L Rewers MJ Klingensmith GJ . Additional autoimmune disease found in 33% of patients at type 1 diabetes onset. Diabetes Care. (2011) 34:1211–3. doi: 10.2337/dc10-1756 21430083 PMC3114477

[6] Mäkimattila S Harjutsalo V Forsblom C Groop P-H . Every fifth individual with type 1 diabetes suffers from an additional autoimmune disease: A Finnish nationwide study. Diabetes Care. (2020) 43:1041–7. doi: 10.2337/dc19-2429 32139386

[7] Biondi B Kahaly GJ Robertson RP . Thyroid dysfunction and diabetes mellitus: Two closely associated disorders. Endocr Rev. (2019) 40:789–824. doi: 10.1210/er.2018-00163 30649221 PMC6507635

[8] Volta U Tovoli F Caio G . Clinical and immunological features of celiac disease in patients with type 1 diabetes mellitus. Expert Rev Gastroenterol Hepatol. (2011) 5:479–87. doi: 10.1586/egh.11.38 21780895

[9] Pociot F Lernmark Å . Genetic risk factors for type 1 diabetes. Lancet. (2016) 387:2331–9. doi: 10.1016/s0140-6736(16)30582-7 27302272

[10] Sanjeevi CB Sedimbi SK Landin-Olsson M Kockum I Lernmark A Swedish Childhood D . Risk conferred by Hla-Dr and Dq for type 1 diabetes in 0-35-year age group in Sweden. Ann N Y Acad Sci. (2008) 1150:106–11. doi: 10.1196/annals.1447.061 19120278

[11] Jerram ST Dang MN Leslie RD . The role of epigenetics in type 1 diabetes. Curr Diabetes Rep. (2017) 17:89. doi: 10.1007/s11892-017-0916-x 28815391 PMC5559569

[12] Rakyan VK Beyan H Down TA Hawa MI Maslau S Aden D . Identification of type 1 diabetes–associated DNA methylation variable positions that precede disease diagnosis. PloS Genet. (2011) 7:e1002300. doi: 10.1371/journal.pgen.1002300 21980303 PMC3183089

[13] Alipoor SD Ahrens A Åkesson J Hillerton T Gustafsson M Lerm M . Epigenetic analyses suggest different pathways during pregnancy for development of type 1 diabetes in children with high versus low‐neutral human leukocyte antigen‐risk. J Internal Med. (2026) 299:570–86. doi: 10.1111/joim.70077 41738077 PMC13061103

[14] Paul DS Teschendorff AE Dang MA Lowe R Hawa MI Ecker S . Increased DNA methylation variability in type 1 diabetes across three immune effector cell types. Nat Commun. (2016) 7:13555. doi: 10.1038/ncomms13555 27898055 PMC5141286

[15] Floess S Freyer J Siewert C Baron U Olek S Polansky J . Epigenetic control of the Foxp3 locus in regulatory T cells. PloS Biol. (2007) 5:e38. doi: 10.1371/journal.pbio.0050038 17298177 PMC1783672

[16] Starskaia I Laajala E Grönroos T Härkönen T Junttila S Kattelus R . Early DNA methylation changes in children developing beta cell autoimmunity at a young age. Diabetologia. (2022) 65:844–60. doi: 10.1007/s00125-022-05657-x 35142878 PMC8960578

[17] Vrachnis N Antonakopoulos N Iliodromiti Z Dafopoulos K Siristatidis C Pappa KI . Impact of maternal diabetes on epigenetic modifications leading to diseases in the offspring. J Diabetes Res. (2012) 2012:538474. doi: 10.1155/2012/538474 23227034 PMC3512252

[18] Ott R Zapardiel-Gonzalo J Kreitmaier P Casteels K Hommel A Kordonouri O . Blood methylome signatures in children exposed to maternal type 1 diabetes are linked to protection against islet autoimmunity. Nat Metab. (2025) 7(11):2236–49. doi: 10.1038/s42255-025-01403-w 41198985 PMC12638243

[19] Ludvigsson J Ludvigsson M Sepa A . Screening for prediabetes in the general child population: Maternal attitude to participation. Pediatr Diabetes. (2001) 2:170–4. doi: 10.1034/j.1399-5448.2001.20405.x 15016182

[20] Nygren M Carstensen J Koch F Ludvigsson J Frostell A . Experience of a serious life event increases the risk for childhood type 1 diabetes: The Abis population-based prospective cohort study. Diabetologia. (2015) 58:1188–97. doi: 10.1007/s00125-015-3555-2 25870022

[21] Russell JT Roesch LF Ördberg M Ilonen J Atkinson MA Schatz DA . Genetic risk for autoimmunity is associated with distinct changes in the human gut microbiome. Nat Commun. (2019) 10:3621. doi: 10.1038/s41467-019-11460-x 31399563 PMC6689114

[22] Assarsson E Lundberg M Holmquist G Bjorkesten J Thorsen SB Ekman D . Homogenous 96-plex pea immunoassay exhibiting high sensitivity, specificity, and excellent scalability. PloS One. (2014) 9:e95192. doi: 10.1371/journal.pone.0095192 24755770 PMC3995906

[23] Ritchie ME Phipson B Wu D Hu Y Law CW Shi W . Limma powers differential expression analyses for RNA-sequencing and microarray studies. Nucleic Acids Res. (2015) 43:e47-e. doi: 10.1093/nar/gkv007 25605792 PMC4402510

[24] Szklarczyk D Kirsch R Koutrouli M Nastou K Mehryary F Hachilif R . The String database in 2023: Protein–protein association networks and functional enrichment analyses for any sequenced genome of interest. Nucleic Acids Res. (2023) 51:D638–46. doi: 10.1093/nar/gkac1000 PMC9825434 36370105

[25] Ahrens AP Dias R Hyötyläinen T Orešič M Triplett EW Ludvigsson J . The inflammatory path toward type 1 diabetes begins during pregnancy. Nat Commun. (2026). doi: 10.1038/s41467-025-67712-6 41501048 PMC12847856

[26] Casagrande V Menini S Internò C Pugliese G Federici M Menghini R . Timp3 overexpression in myeloid lineage alleviates pancreatic damage and confers resistance to the development of type 1 diabetes in the Mlds-induced model. Front Endocrinol. (2024) 14:1297847. doi: 10.3389/fendo.2023.1297847 38313841 PMC10835381

[27] Aamodt KI Powers AC . Signals in the pancreatic islet microenvironment influence β‐cell proliferation. Diabetes Obes Metab. (2017) 19:124–36. doi: 10.1111/dom.13031 28880471 PMC5679109

[28] Nikolova G Jabs N Konstantinova I Domogatskaya A Tryggvason K Sorokin L . The vascular basement membrane: A niche for insulin gene expression and β cell proliferation. Dev Cell. (2006) 10:397–405. doi: 10.1016/j.devcel.2006.01.015 16516842

[29] German MS . Glucose sensing in pancreatic islet beta cells: The key role of glucokinase and the glycolytic intermediates. Proc Natl Acad Sci. (1993) 90:1781–5. doi: 10.1073/pnas.90.5.1781 8446591 PMC45963

[30] Fassah DM Jeong JY Baik M . Hepatic transcriptional changes in critical genes for gluconeogenesis following castration of bulls. Asian-Australas J Anim Sci. (2018) 31:537. doi: 10.5713/ajas.17.0875 29502393 PMC5838326

[31] Lambelet M Terra LF Fukaya M Meyerovich K Labriola L Cardozo AK . Dysfunctional autophagy following exposure to pro-inflammatory cytokines contributes to pancreatic β-cell apoptosis. Cell Death Dis. (2018) 9:96. doi: 10.1038/s41419-017-0121-5 29367588 PMC5833699

[32] Borowiec M Liew CW Thompson R Boonyasrisawat W Hu J Mlynarski WM . Mutations at the Blk locus linked to maturity onset diabetes of the young and β-cell dysfunction. Proc Natl Acad Sci U S A. (2009) 106(34):14460–5. 10.1073/pnas.0906474106 PMC2732833 19667185

[33] Xu F Chen X Hu T Sun R Zhu F Wu X . A novel Blk heterozygous mutation (P. Met121lle) in maturity-onset diabetes mellitus: a case report and literature review. Diabet Med. (2025) 42(6):e15491. 10.1111/dme.15491 39754319

[34] Durand CA Richer MJ Brenker K Graves M Shanina I Choi K . Selective pharmacological inhibition of phosphoinositide 3-kinase p110δ opposes the progression of autoimmune diabetes in non-obese diabetic (NOD) mice. Autoimmunity. (2013) 46(1):62–73. 10.3109/08916934.2012.732130 23039284

[35] Vaitaitis GM Waid DM Yussman MG Wagner DH . CD40-mediated signalling influences trafficking, T-cell receptor expression, and T-cell pathogenesis in the NOD model of type 1 diabetes. Immunology. (2017) 152(2):243–54. 10.1111/imm.12761 PMC5588813 28542921

[36] Chuang ST Alcazar O Watts B Abdulreda MH Buchwald P . Small-molecule inhibitors of the CD40–CD40L costimulatory interaction are effective in pancreatic islet transplantation and prevention of type 1 diabetes models. Front Immunol. (2024) 15:1484425. 10.3389/fimmu.2024.1484425 PMC11599200 39606229

