# Supplementary material for: Early-life epigenetic programming of autoimmune diseases: what is unique to type 1 diabetes
Source: Front Immunol. 2026 Oct 21;17:1946981. doi: 10.3389/fimmu.2026.1946981 (PMC13731104; doi:10.3389/fimmu.2026.1946981)
Supplement: Supplementary file 1 [file DataSheet1.docx]

**Supplementary file 1:**

**Early-life epigenetic programming of autoimmune diseases: what is unique to Type 1 diabetes**

Shamila D. Alipoor^1^, Julia Åkesson ^2,3^, Johnny Ludvigsson ^1,4^*

^1^ Division of Pediatrics, Department of Biomedical and Clinical Sciences, Linköping University, Linköping, Sweden

^2^ Division of Bioinformatics, Department of Physics, Chemistry and Biology, Linköping University, Linköping, Sweden.

^3^ PredictMe AB, Krokgatan 4, 58731 Linköping

^4^ Crown Princess Victoria Children’s Hospital, Linköping University Hospital, Linköping, Sweden

*Corresponding author: [Johnny.Ludvigsson@liu.se](mailto:Johnny.Ludvigsson@liu.se)

**Part 1: Material and Methods**

**Cohort characteristics**

ABIS cohort [1, 2] follows, to present day, 17,055 individuals born between October 1997 and 1999 in Southeastern Sweden. Families were recruited from nine obstetric clinics across all hospitals in the counties of Östergötland, Småland, Blekinge, and Öland. They received oral and written information, and were also offered video-information, and were then asked to join the study. Out of the 21,700 children born in the region, 78.6% of families provided informed consent for their child to participate. Parents completed extensive questionnaires and diaries from the child’s birth through >20 years of age, with ABIS children themselves answering questionnaires at later visits. Cord blood samples were collected from all ABIS children. The ABIS register is connected to the Swedish National Patient Register giving diagnosis of diseases, and to the National Drug Prescription Register which has been used for validation of diagnosis of T1D.

Three groups were included from ABIS cohort: 32 with T1D, as well as individuals

with other autoimmune diseases excluding T1D (NT1D, n=35), identified using ICD-10 codes which comprised celiac disease (K90.0, n=19), psoriasis (L40, n=5), autoimmune thyroiditis

(E06.3, n=4), Crohn’s disease (K50, n=3), juvenile idiopathic arthritis (M08, n=2), and uveitis (H20, n=2). A total of 48 individuals with no recorded history of autoimmune or immune mediated

disease was included as controls.

**Ethical Statement**

Ethical approval for ABIS was obtained by Research Ethics Committees of Faculty of Health Science at Linköping Univ., Ref. 1997/96287 and 2003/03-092 and the Medical Faculty of Lund University Dnr 99227, Dnr 99321. Connection to National Registers was approved (Dnr 03-513 and 2018- 318-32).

**DNA extraction**

Genomic DNA was isolated from whole blood using the QIAamp DNA Mini Kit on the QIAcube Connect automated platform (Qiagen, Germany). DNA concentration was measured with the Quantus Fluorometer and QuantiFluor ONE dsDNA System (E4871, Promega, USA), and DNA quality was evaluated using a NanoDrop ND-1000 spectrophotometer (Thermo Fisher Scientific, USA).

**Methylation array data**

Genomic DNA (250 ng/sample) samples were bisulfite-converted using EZ DNA Methylation Kit (Zymo Research) and profiled using the Illumina MethylationEPIC v1.0 array. BeadChips were scanned on an Illumina NextSeq 550.

**Data preprocessing and quality control**

Methylation signal intensities were extracted from raw IDAT files using the minfi R package (v1.50.0) in R version 4.4.3[3] [4]. During the initial preprocessing stage, signal intensities were normalized and converted to β-values using the intra-sample Beta-Mixture Quantile (BMIQ) normalization[5] procedureand the inter-array Gaussian Mixture Quantile Normalization (GMQN) method[6] implemented in the GMQN R package (v0.1.0) [6].

Probe-level quality control and filtering were applied in the subsequent steps. Probes with missing values in more than 25% of the samples were excluded. Additionally, cross reactive probes, non-CpG probes, probes mapping to known single nucleotide polymorphisms (SNPs), and probes located on sex chromosomes (X and Y) were discarded using the ChAMP R package (v2.34.0) [4] . Remaining missing values were imputed using k-nearest neighbor (kNN) imputation via the scikit-learn Python package[7]

To identify and correct technical artifacts, singular value decomposition (SVD) analysis was performed using a modified version of the SVD function from the ChAMP R package. Major sources of variation significantly associated (P ≤ 0.05 ) with SVD components explaining

> 3% of the variance, such as slide and array effects, were adjusted using the ComBat algorithm from the sva R package (v3.52.0) [8]. A follow-up SVD analysis confirmed successful artifact removal.

Finally, data quality was comprehensively evaluated using density plots of β-value distributions. Principal component analysis (PCA) was performed to visualize sample clustering and confirm preprocessing efficiency.

**Differential DNA methylation analysis**

Differentially methylated CpGs (DMCs) between groups were identified using the *limma R* package (v3.60.6) [9]. For each CpG, linear models were fitted adjusting for sex and cell type proportions that differed significantly between groups (P ≤ 0.05). Empirical Bayes moderation was applied to stabilize variance estimation, and differential methylation was assessed using moderated t-statistics. We applied a *P value* cutoff of 0.05 and a |logFC| cutoff of 0.05 to determine whether a CpG was differentially methylated [10, 11]. The identified DMCs were mapped to corresponding differentially methylated genes (DMGs) using Illumina EPIC array annotations *ilm10b4.hg19*[12]. The results were visualized as heatmaps using *ComplexHeatmap* package (v2.10.0)[10, 11] and as volcano plots using *ggplot2*[13] in R(v3.4.4).

**Pathway enrichment analysis**

KEGG pathway enrichment analysis of differentially methylated CpGs was performed using the probe-aware gometh function implemented in the missMethyl R package. The post-quality-control CpGs that entered the differential methylation analyses were used as the background probe set. The Wallenius noncentral hypergeometric test was used to account for differences in probe numbers across genes. The analysis was performed with fract.counts = TRUE to account for CpGs annotated to multiple genes. CpGs meeting the nominal significance threshold of P ≤0.05 and |Δβ|≥ 0.05 were used as the significant input set.

**Serum proteomics and Statistical analysis**

Circulating protein levels in neonatal serum were measured using the Olink Explore 384 Inflammation and Oncology panels (Olink Proteomics, Uppsala, Sweden), deployed on the next-generation sequencing-based Olink Explore platform[14] . Protein abundance was expressed as Normalized Protein eXpression (NPX) values on a log2 scale, covering 727 assays following manufacturer-specified quality control procedures and normalization protocols. Differences in protein abundance between groups were assessed using linear models implemented in the limma package, with sex and pool included as covariates, followed by empirical Bayes moderation of variance estimates. Proteins were considered differentially abundant at a nominal P-value ≤ 0.05 and an absolute median ΔNPX ≥ 0.2 .

**Integration and network analysis of epigenetic-proteomic data**

To investigate cross-layer molecular interactions, differentially methylated genes (DMGs) from cord blood DNA methylation data and differentially abundant serum proteins were integrated using a network-based approach. DMGs were obtained from our previously published epigenome-wide analysis of the ABIS cohort [1, 15], in which differential methylation was assessed using the limma R package[9]. CpG sites were considered differentially methylated at nominal p-value thresholds (P ≤ 0.05) and |logFC| ≥ 0.05. The resulting list of DMGs was used as input for the integrative network analysis. Serum proteins were selected based on effect size (|ΔNPX| ≥ 0.2) and nominal p ≤ 0.05.

The molecular inputs for network analysis were derived from the quality-controlled methylation and proteomic datasets. The proteomic component comprised the 727 Olink assays that passed quality control, while the methylation component comprised CpG sites retained after preprocessing and quality control, from which DMGs were derived for network construction. . The resulting DMGs and differentially abundant proteins from each comparison were combined and mapped to the STRING database (version 12.0) (24) for protein–protein interaction (PPI) retrieval. Unmapped identifiers were excluded (removeUnmappedRows = TRUE). Only high-confidence interactions (combined score ≥ 0.7) were retained. The resulting integrated epigenetic–proteomic interaction network was constructed separately for each comparison.

**Statistics:**

Differential DNA methylation was evaluated with limma using moderated t-statistics, and CpG sites were considered differentially methylated at P ≤ 0.05 and |logFC| ≥ 0.05. For pathway enrichment, significance was set at P ≤ 0.05. For Olink, Group differences were evaluated using Wilcoxon rank-sum tests, with p-values ≤ 0.05 and |ΔNPX| ≥ 0.2 were considered differentially abundant.

**Part 2: Differential proteomic analysis of neonatal serum across study comparisons.**

Representative boxplots show normalized protein expression (NPX) levels for selected proteins across the three study comparisons: future type 1 diabetes (T1D) versus future non-type 1D- autoimmune diseases (NT1D), T1D versus healthy controls, and NT1D versus healthy controls. Proteins were selected based on biological relevance and their contribution to the integrative epigenetic–proteomic analyses. The p-value and ΔNPX (difference in normalized protein expression) from the differential abundance analysis are shown for each protein. Volcano plots display differentially abundant proteins (nominal p < 0.05 and |ΔNPX| ≥ 0.2), with proteins passing FDR < 0.05 are indicated by triangles. KEGG pathway enrichment analysis was performed using differentially abundant proteins with nominal p < 0.05.

**T1D vs NT1D:**

**T1D vs Control:**

**NT1D vs Control:**
