# Supplementary material for: Early-life epigenetic programming of autoimmune diseases: what is unique to type 1 diabetes
Source: Front Immunol. 2026 Oct 21;17:1946981. doi: 10.3389/fimmu.2026.1946981 (PMC13731104; doi:10.3389/fimmu.2026.1946981)
Supplement: Supplementary file 2 [file Table3.docx]

**Supplementary Table 3. Sensitivity analysis of crosstalk networks with the text-mining evidence channel excluded.**

For each pairwise comparison, the original STRING v12 crosstalk network (all seven evidence channels, combined score ≥ 700) was compared to a network built after excluding the text-mining evidence channel. The remaining six channels (neighborhood, gene fusion, co-occurrence, co-expression, experiments, databases) were recombined using STRING's standard prior-removal and integration procedure, applying the same confidence threshold. Jaccard similarity was calculated as the number of shared nodes/interactions divided by the total number of unique nodes/interactions across both networks. In all three comparisons, every node and interaction present in the text-mining-excluded network was also present in the original network (0 text-mining-OFF-only interactions), indicating that exclusion of text mining did not introduce spurious associations.

| **Comparison** | **Original nodes** | **Text-mining-OFF nodes** | **Node Jaccard** | **Original interactions** | **Text-mining-OFF interactions** | **Edge Jaccard (% retained)** |
| --- | --- | --- | --- | --- | --- | --- |
| **T1D vs NT1D** | 103 | 47 | 0.456 | 118 | 40 | 0.339 (33.9%) |
| **T1D vs Control** | 62 | 25 | 0.403 | 62 | 19 | 0.306 (30.6%) |
| **NT1D vs Control** | 69 | 26 | 0.377 | 63 | 16 | 0.254 (25.4%) |

*Abbreviations: T1D, type 1 diabetes; NT1D, non-progressor/non-converted to T1D; Jaccard, Jaccard similarity index (intersection/union).*
